# Sustained Complete Remission After a Single Cycle of R-CHOP in Extranodal Diffuse Large B-Cell Lymphoma of the Mandible: A Case Report

**DOI:** 10.7759/cureus.105242

**Published:** 2026-03-14

**Authors:** Rodrigo Furlan Silva Fabri, Suyog Patel, Rodolfo Myronn De Melo Rodrigues, Jesus Gomez

**Affiliations:** 1 Internal Medicine, Texas Tech University Health Sciences Center El Paso, El Paso, USA; 2 Hematology and Medical Oncology, University Medical Center of El Paso, El Paso, USA

**Keywords:** case report, diffuse large b-cell lymphoma, immune-mediated tumour regression, mandibular lymphoma, r-chop chemotherapy, spontaneous remission

## Abstract

Diffuse large B-cell lymphoma (DLBCL) is an aggressive yet potentially curable malignancy when treated with standard multi-cycle R-CHOP (Rituximab, Cyclophosphamide, Doxorubicin hydrochloride (Hydroxydaunorubicin), Vincristine sulfate (Oncovin), and Prednisolone/Prednisone) chemo-immunotherapy. Durable remission after incomplete or abbreviated therapy is exceptionally uncommon. We report the case of a 38-year-old man diagnosed with extranodal DLBCL of the mandible who received one cycle of R-CHOP before discontinuing therapy due to loss of insurance coverage. He was lost to follow-up and later re-presented with mild local symptoms. Restaging with contrast-enhanced computed tomography of the neck, chest, abdomen, and pelvis, along with magnetic resonance imaging of the brain and face, demonstrated no evidence of residual, recurrent, or disseminated disease. Laboratory studies, including complete blood count, comprehensive metabolic panel, and lactate dehydrogenase, were within normal limits. The patient has been in sustained complete clinical and radiologic remission for 24 months after a single cycle of R-CHOP. This case highlights the rarity of durable remission after minimal therapy in DLBCL and supports further investigation into biologic mechanisms associated with exceptional responses.

## Introduction

Diffuse large B-cell lymphoma (DLBCL) is the most common subtype of non-Hodgkin lymphoma, accounting for approximately 30-40% of cases worldwide, and is characterized by aggressive clinical behavior and significant biological heterogeneity [1]. Standard first-line therapy consists of rituximab in combination with cyclophosphamide, doxorubicin hydrochloride (hydroxydaunorubicin), vincristine (Oncovin), and prednisone (R-CHOP), administered over multiple cycles, with curative intent in a substantial proportion of patients [1]. Durable remission and long-term survival are strongly associated with completion of planned therapy. Extranodal disease is frequently encountered in DLBCL; however, primary mandibular involvement is uncommon among extranodal presentations and may manifest with pain, swelling, and destructive bony changes, often prompting concern for aggressive local pathology.

Although DLBCL is generally highly chemosensitive, sustained complete remission following incomplete or abbreviated treatment is exceedingly rare. Spontaneous regression of malignant tumors has been recognized for centuries and remains a poorly understood phenomenon in oncology [2]. Contemporary literature has documented rare cases of spontaneous or near-spontaneous remission across multiple cancer types, suggesting a potential role for immune-mediated tumor control [3,4]. Within DLBCL, durable remission after incomplete or minimal therapy remains exceptionally rare and is largely limited to isolated case reports. Spontaneous regression refers to tumor regression in the absence of antineoplastic therapy, whereas durable remission after minimal therapy may represent a distinct, therapy-triggered phenomenon.

Emerging evidence indicates that activation of innate and adaptive immune pathways may contribute to tumor regression in select cases. Chemotherapy-induced immunogenic cell death has been proposed as a mechanism by which cytotoxic therapy may enhance antitumor immune responses through antigen release and activation of damage-associated molecular patterns [5]. These observations parallel modern immunotherapeutic strategies that aim to harness host immunity for durable disease control.

Despite advances in treatment, outcomes for patients with relapsed or refractory DLBCL remain poor, underscoring the aggressive nature of the disease and the importance of adequate initial therapy [6]. Rare reports have described spontaneous or prolonged remission of DLBCL following minimal intervention, including cases occurring after biopsy alone or incomplete treatment, further highlighting the potential contribution of immune mechanisms [7,8].

We report a highly unusual case of extranodal DLBCL of the mandible achieving sustained complete clinical and radiologic remission 24 months after receiving only a single cycle of R-CHOP chemotherapy. This case adds to the limited body of literature describing durable remission after minimal therapy and raises important questions regarding immune-mediated tumor control in aggressive lymphomas.

## Case presentation

**Figure 1:**
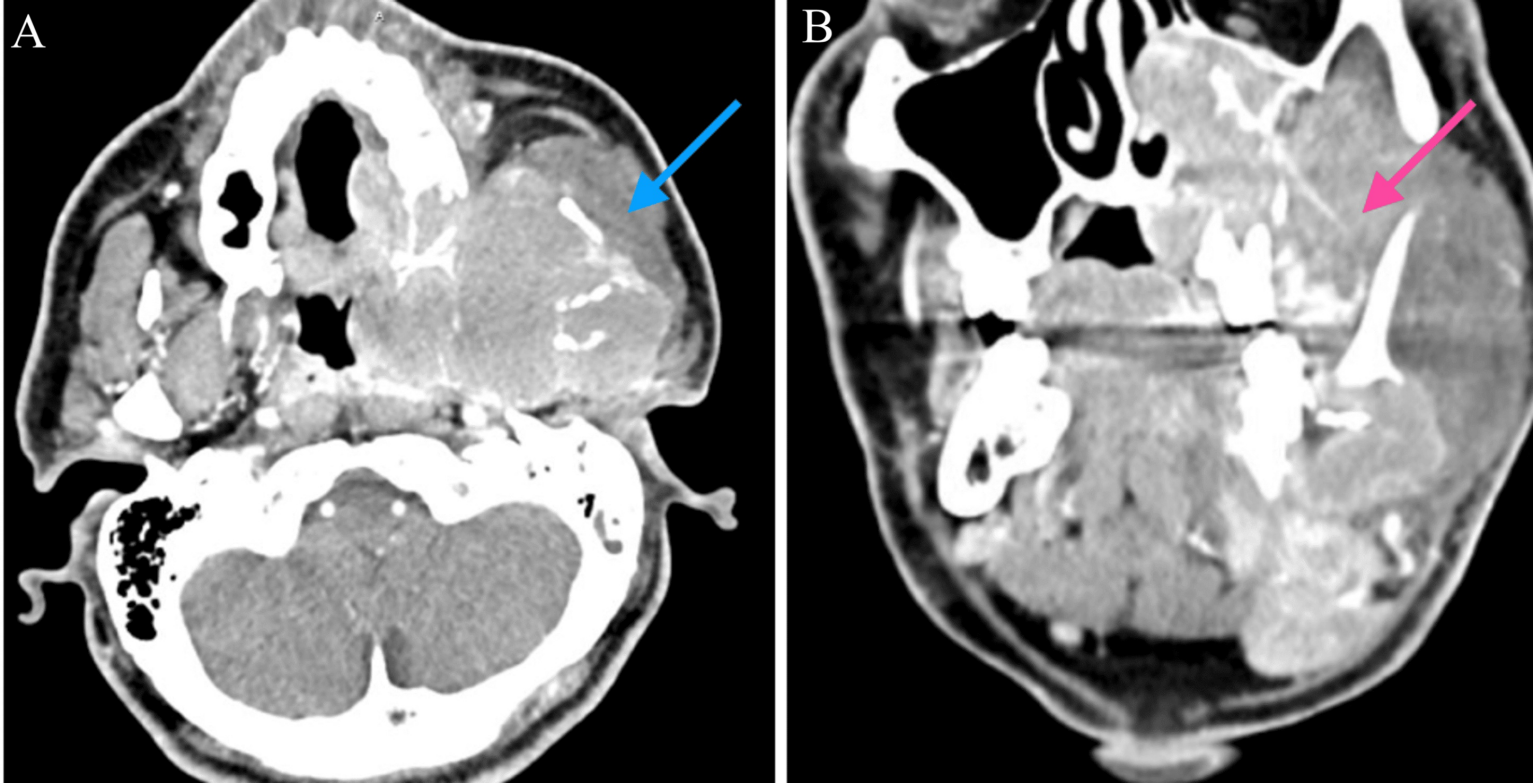
CECT of the face demonstrating extensive destructive left mandibular mass at initial presentation (A) Axial view shows a large heterogeneous soft-tissue mass centered in the left mandibular region with aggressive local extension (blue arrow). (B) Coronal view demonstrates the superior–inferior extent of the lesion with involvement of adjacent maxillofacial structures (pink arrow), consistent with extranodal diffuse large B-cell lymphoma with locally destructive features. CECT: contrast-enhanced computed tomography

A 38-year-old man with no significant past medical history presented in May 2023 with progressive pain and swelling involving the left side of his face. Contrast-enhanced computed tomography (CECT) revealed a large destructive mass measuring approximately 10 × 6 × 10 cm involving the left mandible, maxilla, and skull base, with extension into the parapharyngeal space, associated bone erosion, and regional lymphadenopathy (Figure 1).

**Figure 2:**
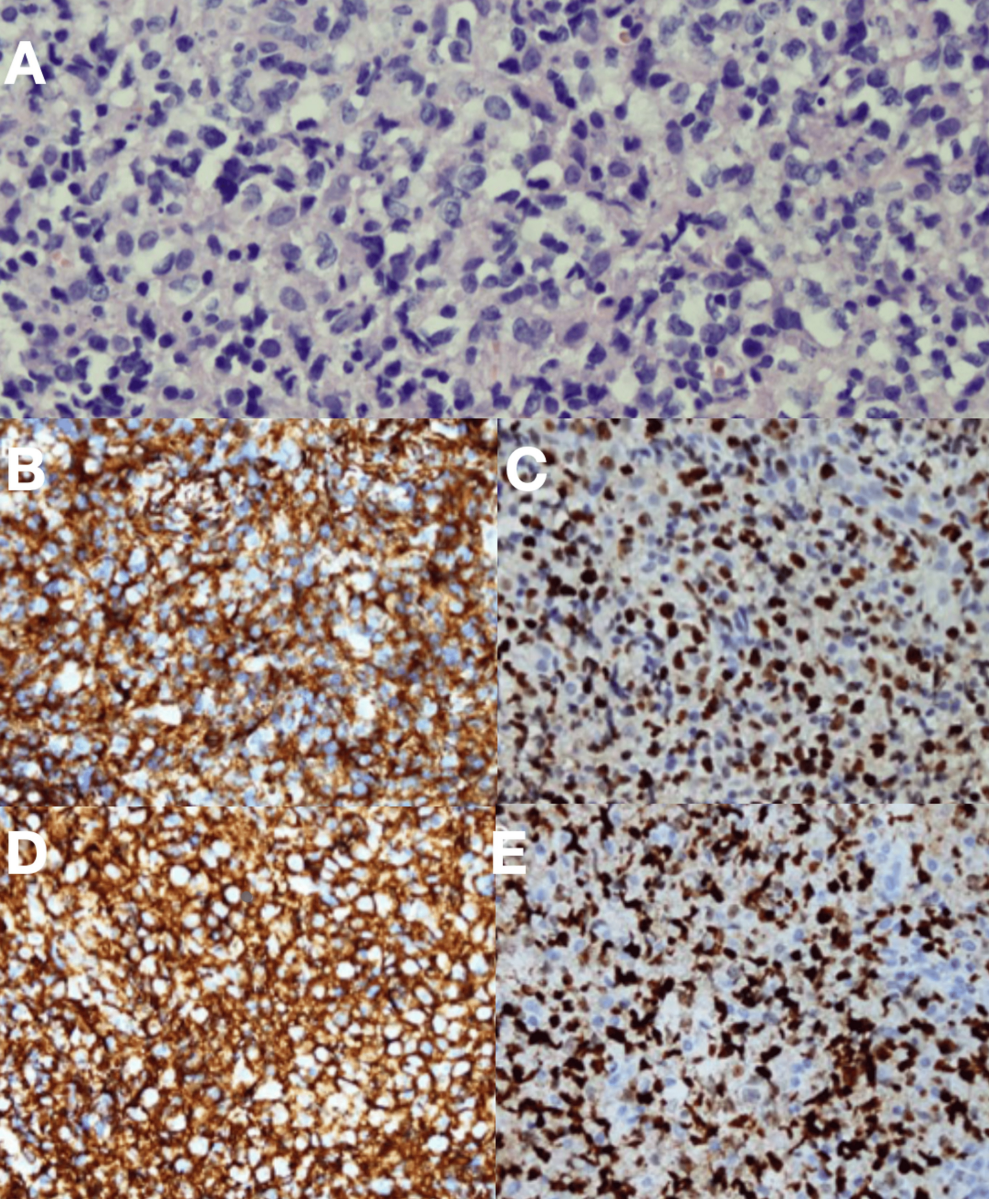
Histopathologic and immunohistochemical characterization of mandibular biopsy confirming diffuse large B-cell lymphoma (A) Hematoxylin and eosin (H&E) stain demonstrating diffuse sheets of large atypical lymphoid cells with vesicular chromatin, prominent nucleoli, and moderate cytoplasm, consistent with diffuse large B-cell lymphoma. (B) CD20 immunohistochemical staining showing strong diffuse membranous positivity, confirming B-cell lineage. (C) BCL6 immunohistochemical staining demonstrating nuclear positivity in tumor cells. (D) CD10 immunohistochemical staining demonstrating tumor cell positivity, supporting germinal center phenotype. (E) Ki-67 immunohistochemical staining demonstrating a high proliferative index (approximately 80%), consistent with aggressive tumor biology.

Histopathologic examination of a biopsy (Figure 2) specimen confirmed diffuse large B-cell lymphoma. Immunohistochemistry demonstrated positivity for CD20, CD10, BCL6, and cyclin D1, with a high proliferative index (Ki-67 approximately 80%). Fluorescence in situ hybridisation identified a c-MYC rearrangement and BCL6 copy gain, without evidence of BCL2 rearrangement.

The patient received one cycle of R-CHOP chemotherapy, which he tolerated without complications. Owing to financial hardship and loss of insurance coverage, he was unable to continue therapy and was subsequently lost to medical follow-up.

**Figure 3:**
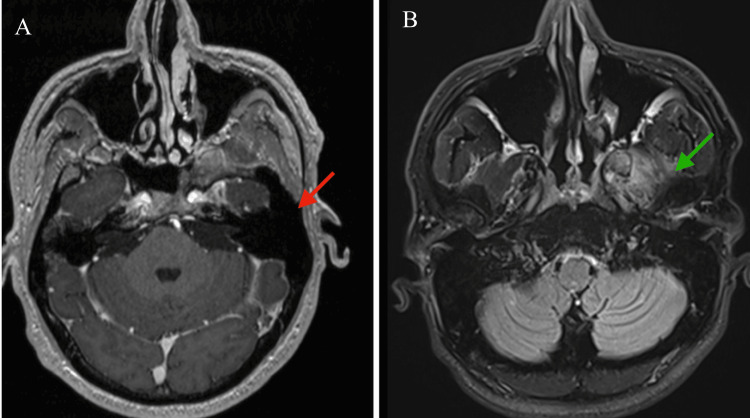
Axial contrast-enhanced MRI of the face demonstrating sustained complete remission of extranodal diffuse large B-cell lymphoma (A) T1-weighted post-contrast MRI (axial view) obtained at the same anatomic level demonstrating no residual or recurrent enhancing lesion (red arrow). (B) T1-weighted post-contrast MRI (axial view) obtained at the same anatomic level as the initial CT, demonstrating fibrosis with no residual or recurrent enhancing lesion (green arrow). No biopsy of the fibrotic-appearing area was performed.

Upon re-presentation 24 months after the single R-CHOP cycle, he reported mild left-sided facial pressure, intermittent blurred vision, and imbalance while walking, without fever, weight loss, night sweats, or other constitutional symptoms. Physical and neurological examinations were unremarkable. Repeat contrast-enhanced imaging, including MRI of the face and brain, demonstrated no evidence of residual or recurrent lymphoma and no evidence of central nervous system involvement (Figure 3). Restaging with CECT of the neck, chest, abdomen, and pelvis demonstrated no additional sites of disease. Laboratory studies, including complete blood count, comprehensive metabolic panel, and lactate dehydrogenase, were within normal limits, supporting sustained complete clinical and radiologic remission.

## Discussion

This case represents an extraordinary example of sustained complete remission of DLBCL following only a single cycle of standard R-CHOP therapy. Although DLBCL is an aggressive malignancy, it is also highly chemosensitive, with durable remission and cure typically requiring completion of multi-cycle treatment [1]. Durable remission after incomplete therapy is therefore exceptionally uncommon. This outcome is particularly striking given the presence of adverse molecular features, including c-MYC rearrangement with BCL6 copy gain, which are generally associated with aggressive disease biology and inferior outcomes. Sustained remission after a single R-CHOP cycle in this setting is therefore extraordinary.

Spontaneous or near-spontaneous regression of malignancy has been recognized for centuries, with historical and modern reports describing tumor regression following immune activation, infection, or inflammation [2-4]. These events are rare but appear disproportionately represented among hematological malignancies, particularly aggressive lymphomas [7,8].

It is unlikely that a single cycle of R-CHOP alone resulted in complete cytotoxic eradication of the tumor burden. Instead, chemotherapy-induced immunogenic cell death may have served as a trigger for immune-mediated tumor control. Cytotoxic therapy can promote tumor antigen release, activation of damage-associated molecular patterns, and stimulation of innate immune pathways, facilitating downstream adaptive immune responses [5]. This concept aligns with contemporary immunotherapeutic strategies that seek to exploit similar mechanisms [6].

The sustained remission observed here suggests that, in rare circumstances, host immune responses may contribute to durable control despite unfavorable molecular characteristics. Alternative explanations should also be considered, including initial staging uncertainty, sampling limitations, and tumor heterogeneity, transient tumor collapse after minimal therapy, occult residual disease below imaging detection thresholds, and variability in host immune surveillance. In addition, the absence of PET-CT limits confirmation of complete metabolic remission and introduces uncertainty regarding the depth of response.

Beyond biological considerations, this case also highlights systemic barriers to cancer care. Interruption of potentially curative therapy due to loss of insurance coverage remains a significant challenge. Although the favorable outcome in this case is exceptional and should not be generalized, it underscores the complex and sometimes unpredictable interplay between therapy, immunity, and tumor biology.

In summary, this case adds to the limited body of literature describing durable remission of DLBCL after minimal therapy [2-5,7,8]. Such rare observations provide valuable insight into immune-mediated tumor control and support further investigation into therapeutic strategies that harness innate and adaptive immunity in aggressive lymphomas.

## Conclusions

Sustained complete remission of extranodal diffuse large B-cell lymphoma following a single cycle of R-CHOP chemotherapy is extraordinarily uncommon and stands in contrast to the established paradigm that multi-cycle chemo-immunotherapy is required for durable disease control. The prolonged clinical and radiologic remission observed in this case, despite high-risk disease features and early treatment discontinuation, highlights the remarkable heterogeneity of lymphoma biology and therapeutic response.

This case is hypothesis-generating and suggests that limited chemotherapy exposure may, in rare circumstances, be associated with prolonged disease control through mechanisms beyond direct cytotoxicity, including the possibility of therapy-triggered immune-mediated tumor regression; however, this mechanism is not demonstrated in this report. Chemotherapy-induced immunogenic cell death is a biologically plausible mechanism that could enhance tumor antigen presentation and host immune activation, but this was not directly demonstrated in this case. Although such outcomes should not be generalized or used to justify abbreviated therapy, they provide valuable insight into the potential interplay between cytotoxic treatment and immune surveillance.

Furthermore, this case underscores the importance of long-term follow-up and comprehensive restaging in patients who discontinue therapy prematurely, as unexpected outcomes may occur. From a broader perspective, rare instances of spontaneous or near-spontaneous remission offer a unique opportunity to explore immune mechanisms that could be therapeutically harnessed in aggressive lymphomas. Further investigation into the biological and immunologic factors contributing to such exceptional responses may inform future treatment strategies aimed at enhancing durable remission while minimizing treatment-related toxicity.
